# Reply to Sergent et al. Comment on “Balwierz et al. Potential Carcinogens in Makeup Cosmetics. *Int. J. Environ. Res. Public Health* 2023, *20*, 4780”

**DOI:** 10.3390/ijerph21020161

**Published:** 2024-01-31

**Authors:** Radosław Balwierz, Paweł Biernat, Agata Jasińska-Balwierz, Dawid Siodłak, Anna Kusakiewicz-Dawid, Anna Kurek-Górecka, Paweł Olczyk, Wioletta Ochędzan-Siodłak

**Affiliations:** 1 Faculty of Chemistry, University of Opole, 45-052 Opole, Poland; 2 Department of Drug Forms Technology, Faculty of Pharmacy, Wroclaw Medical University, 50-556 Wroclaw, Poland; 3 Department of Pharmacology, Academy of Silesia, 40-555 Katowice, Poland; 4 Department of Community Pharmacy, Faculty of Pharmaceutical Sciences in Sosnowiec, Medical University of Silesia in Katowice, Kasztanowa 3, 41-200 Sosnowiec, Poland

Comments by Sergent et al. (2023) [1] are relevant and provide important information regarding the identity and nomenclature of “silica”. These comments serve as a valuable addition to the data presented in our publication. The position expressed by Sergent et al. offers an opportunity to strengthen and expand the discussion. It is important to emphasize that the primary objective of our published work was to bring attention to the issues of potentially carcinogenic substances in cosmetics, which are not entirely clear and obvious. Through reviewing the literature and highlighting inaccuracies and doubts, we aimed to shed light on important considerations for the use of cosmetics, especially considering the often long-term relationship between the skin and makeup, for which there is a lack of data within the literature.

In relation to the position presented by Sergent et al. [1] regarding silica nomenclature, we, as the authors of the publication, concur with their clear stance. However, we would like to point out and clearly emphasize that the amorphous silica in our work is not a carcinogenic material and that we define its importance for cosmetic products. We specifically mention that silica is a commonly used filler in cosmetics, which enhances the application properties of powder products, improves adhesion, and facilitates even distribution. In cosmetics designed for oily and combination skin, silica is employed as a matting agent. We have indicated that silicon dioxide (silica) is a permissible ingredient in cosmetics without quantitative restrictions. The information we have provided aligns with the data available in the Cosing database, which states that silica has the CAS number 7631-86-9/112945-52-5/60676-86-0. The aforementioned information is clearly presented in our article because we relied on information sourced from the publicly available Cosing database.

Silica was included in our review due to its widespread use in cosmetics, and, as authors, we acted in good faith to objectively present our position and perspective. However, we acknowledge that further research and verification are necessary to fully understand its potential carcinogen effects. Our chapter in the publication should be regarded as a contribution to the ongoing discussion rather than a definitive conclusion. Furthermore, we presented various positions published by other authors in our review. The information regarding silica was presented without any conclusion in the paper, and no definitive conclusion was drawn regarding its safety. We maintained the overall focus on the issue of safety and did not question it. We want to emphasize once again that the use of amorphous silica is considered safe, and there are no quantitative restrictions on its use, as indicated in the text.

Furthermore, there is a typo within the abstract, and we meant to write “IARC” instead of “IRAC.”

We would like to clarify that the purpose of our work was not to review the various forms of silica available. Therefore, the comment provided by Sergent et al. [1] serves as a valuable supplement to the information presented in our paper. We agree with Sergent et al. that crystalline and amorphous silica have different toxicological profiles. However, we find the reference cited by the authors, specifically https://www.sterlingminerals.com/is-silica-powder-63safein-mineral-makeup-products (accessed on 24 July 2023), to be non-binding and unconvincing due to the nature of the citation being presented in a blog form rather than as a peer-reviewed scientific publication. When conducting specific searches using key phrases in medical databases, we come across a significant amount of inconsistent information. Inconsistencies primarily arise from the multitude of names for silica varieties. Differences in color and physical properties along with the hybridization of forms or regional variations can also lead to nomenclature confusion. Regarding cosmetics, much of the confusion stems from the designation in the Cosing database, as mentioned earlier. Additionally, when searching for information in medical databases using keywords such as “silica,” one can encounter data on various types of silica, further contributing to the confusion. However, we want to emphasize that we clearly indicate that it is crystalline silica and not amorphous silica, which is associated with lung cancer. We referred to the “nano” forms of silica, the inhalation of which cannot be completely ruled out during application. Sergent et al. suggests that makeup products cannot potentially be inhaled, but this cannot be entirely dismissed in the case of dry powders. Although the risk may be minimal, the long-term effects of exposure are still unknown. Additionally, considering the long-lasting relationship between the skin and makeup, it is worth noting that nanoparticles, as indicated by Huang’s extensive review paper [2], can potentially induce cardiovascular toxicity. Silica nanoparticles seem to be safe for the eyes. However, concerning liver toxicity, Huang et al. note that nearly all sizes of silica nanoparticles have been found to cause fibrosis in experimental animals. Data on gastrointestinal tract toxicity are also inconclusive, suggesting that nanoparticles with a size above 100 nm can potentially be toxic. Therefore, complete indifference to nanoparticles cannot be ruled out, and the potential formation of nanoparticles during the application of loose powders cannot be completely ignored.

It is important to note that nanoparticles should be clearly labeled on cosmetics packaging.

Both our position and that of Sergent et al. are consistent, and we agree with the authors that the nomenclature of silica on cosmetics packaging should be clarified to avoid potential misleading information. We want to emphasize once again that we do not make a definitive statement about the carcinogenicity of silica in any part of our work. The title of the chapter itself is presented with a question mark, indicating our intention to contribute to the discussion. Therefore, we consider that the comments made by Sergent et al. are a valuable addition to the position we have presented.
